# Diaqua­bis(benzyl­oxyacetato)copper(II)

**DOI:** 10.1107/S1600536808010593

**Published:** 2008-04-23

**Authors:** Sheng-Li Sun, Chun-Liang Chen, Chang-Sheng Gu, Weng-Dong Song, Xiao-Min Hao

**Affiliations:** a Monitoring Center of Marine Resources and the Environment, Guangdong Ocean University, Zhanjiang 524088, People’s Republic of China; b Department of Applied Chemistry, Guangdong Ocean University, Zhanjiang 524088, People’s Republic of China

## Abstract

In the title mononuclear complex, [Cu(C_9_H_9_O_3_)_2_(H_2_O)_2_], the Cu^II^ ion, located on an inversion center, is hexa­coordinated by four O atoms from two benzyl­oxyacetate ligands [Cu—O = 1.9420 (14) and 2.2922 (14) Å] and two water mol­ecules [Cu—O = 2.0157 (15) Å] in a distorted octa­hedral geometry. In the crystal structure, inter­molecular O—H⋯O hydrogen bonds link the mol­ecules into layers parallel to the *bc* plane.

## Related literature

For general background, see: Eddaoudi *et al.* (2005 ▶).
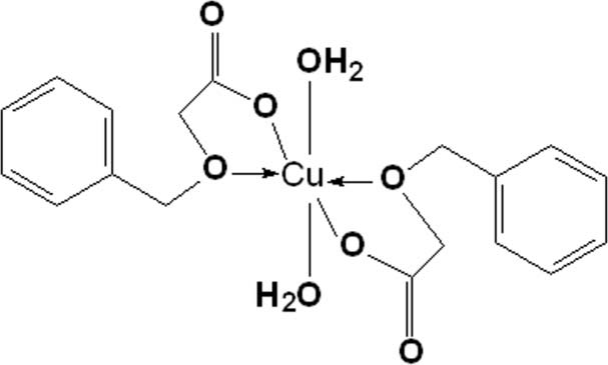


## Experimental

### 

#### Crystal data

[Cu(C_9_H_9_O_3_)_2_(H_2_O)_2_]
*M* *_r_* = 429.91
Monoclinic, 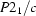

*a* = 11.8847 (4) Å
*b* = 7.1509 (2) Å
*c* = 11.6564 (5) Å
β = 110.283 (3)°
*V* = 929.21 (6) Å^3^
*Z* = 2
Mo *K*α radiation
μ = 1.22 mm^−1^
*T* = 296 (2) K
0.32 × 0.24 × 0.18 mm

#### Data collection

Bruker P4/APEXII diffractometer
Absorption correction: multi-scan (*SADABS*; Sheldrick, 1996 ▶) *T*_min_ = 0.711, *T*_max_ = 0.803
7991 measured reflections
2144 independent reflections
1606 reflections with *I* > 2σ(*I*)
*R*_int_ = 0.035

#### Refinement

*R*[*F* ^2^ > 2σ(*F* ^2^)] = 0.034
*wR*(*F* ^2^) = 0.082
*S* = 1.03
2144 reflections
130 parameters
3 restraints
H atoms treated by a mixture of independent and constrained refinement
Δρ_max_ = 0.40 e Å^−3^
Δρ_min_ = −0.57 e Å^−3^

### 

Data collection: *APEX2* (Bruker, 2004 ▶); cell refinement: *SAINT* (Bruker, 2004 ▶); data reduction: *SAINT*; program(s) used to solve structure: *SHELXS97* (Sheldrick, 2008 ▶); program(s) used to refine structure: *SHELXL97* (Sheldrick, 2008 ▶); molecular graphics: *SHELXTL* (Sheldrick, 2008 ▶); software used to prepare material for publication: *SHELXL97*.

## Supplementary Material

Crystal structure: contains datablocks I, global. DOI: 10.1107/S1600536808010593/cv2398sup1.cif

Structure factors: contains datablocks I. DOI: 10.1107/S1600536808010593/cv2398Isup2.hkl

Additional supplementary materials:  crystallographic information; 3D view; checkCIF report

## Figures and Tables

**Table 1:** Hydrogen-bond geometry (Å, °)

*D*—H⋯*A*	*D*—H	H⋯*A*	*D*⋯*A*	*D*—H⋯*A*
O1*W*—H1*W*1⋯O2^i^	0.839 (9)	1.978 (12)	2.796 (2)	165 (2)
O1*W*—H1*W*2⋯O2^ii^	0.83 (2)	1.964 (10)	2.788 (2)	173 (2)

Symmetry codes: (i) 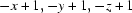; (ii) 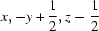.

**Fig. 1.**
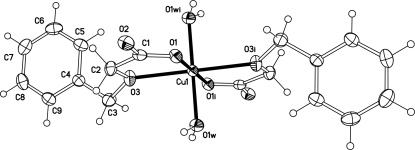
The molecular structure of (I) showing the atomic numbering and 30% probability displacement ellipsoids [symmetry code: (i) -x+1, -y, -z+1].

## supplementary crystallographic information

### Comment

Current interests in supramolecular chemistry are rapidly expanding for their intriguing architectures and potential applications (Eddaoudi *et al.*, 2005). The organic aromatic carboxylate ligand, benzyloxyacetate, has various coordination modes and can link metal centres through carboxylate groups or/and benzyloxy group into different extended architectures. Therefore, benzyloxyacetate can be considered as a good candidate to construct various metal-organic complexes. Herein we report the crystal structure of the title mononuclear complex of benzyloxyacetate, [Cu(C_9_H_9_O_3_)_2_(H_2_O)_2_], (I).

As illustrated in Fig. 1, the Cu^II^ ion lies on an inversion center and displays an octahedral geometry defined by four carboxylate O atoms from two different benzyloxyacetate ligands and two water molecules. The Cu—O and Cu—Ow bond lengths are 1.942 (1), 2.292 (1) and 2.016 (2) Å, respectively. The characteristic *C*—*O*(carboxylate) bond lengths suggest electron localization of the carboxylate groups of the anionic ligands. In the crystal structure, intermolecular hydrogen bonds (Table 1) give rise to a supramolecular structure.

### Experimental

The ligand, benzyloxyacetic acid was commercially available and used without further purification. The title complex was prepared by the addition of Cu(Ac)_2_.H_2_O (4.00 g, 20 mmol) to a hot aqueous solution of benzyloxyacetic acid (1.66 g, 10 mmol); the pH was adjusted to 6 with 0.1*M* sodium hydroxide. The solution was allowed to evaporate at room temperature. Blue prismatic crystals were separated from the filtered solution after several days. C&H analysis. Calc. for C_18_H_22_CuO_8_: C 50.28, H 5.16%. Found: C 50.26, H 5.17%.

### Refinement

The C-bound H atoms were placed in calculated positions, with C—H = 0.93 or 0.97 Å, and were refined in the riding-model approximation, with *U*_iso_(H) = 1.2*U*_eq_(C). The H atoms of the water molecule were located in a difference Fourier map and refined with bond restrint O—H = 0.84 (2) Å in the riding-model approximation, with *U*_iso_(H) = 1.5*U*_eq_(O).

### Crystal data

**Table:**

[Cu(C_9_H_9_O_3_)_2_(H_2_O)_2_]	*F*_000_ = 446
*M**_r_* = 429.91	*D*_x_ = 1.536 Mg m^−^^3^
Monoclinic, *P*2_1_/*c*	Mo *K*α radiation λ = 0.71073 Å
Hall symbol: -P 2ybc	Cell parameters from 7991 reflections
*a* = 11.8847 (4) Å	θ = 1.8–27.6º
*b* = 7.1509 (2) Å	µ = 1.22 mm^−^^1^
*c* = 11.6564 (5) Å	*T* = 296 (2) K
β = 110.283 (3)º	Prism, blue
*V* = 929.21 (6) Å^3^	0.32 × 0.24 × 0.18 mm
*Z* = 2	

### Data collection

**Table:**

Bruker P4 diffractometer	2144 independent reflections
Radiation source: fine-focus sealed tube	1606 reflections with *I* > 2σ(*I*)
Monochromator: graphite	*R*_int_ = 0.035
Detector resolution: 10.000 pixels mm^-1^	θ_max_ = 27.6º
*T* = 296(2) K	θ_min_ = 1.8º
ω scans	*h* = −15→15
Absorption correction: multi-scan(SADABS; Sheldrick, 1996)	*k* = −9→8
*T*_min_ = 0.711, *T*_max_ = 0.803	*l* = −15→10
7991 measured reflections	

### Refinement

**Table:**

Refinement on *F*^2^	Secondary atom site location: difference Fourier map
Least-squares matrix: full	Hydrogen site location: inferred from neighbouring sites
*R*[*F*^2^ > 2σ(*F*^2^)] = 0.034	H atoms treated by a mixture of independent and constrained refinement
*wR*(*F*^2^) = 0.082	*w* = 1/[σ^2^(*F*_o_^2^) + (0.0429*P*)^2^] where *P* = (*F*_o_^2^ + 2*F*_c_^2^)/3
*S* = 1.03	(Δ/σ)_max_ < 0.001
2144 reflections	Δρ_max_ = 0.40 e Å^−^^3^
130 parameters	Δρ_min_ = −0.57 e Å^−^^3^
3 restraints	Extinction correction: none
Primary atom site location: structure-invariant direct methods	

### Fractional atomic coordinates and isotropic or equivalent isotropic displacement parameters (Å^2^)

**Table:**

	*x*	*y*	*z*	*U*_iso_*/*U*_eq_	
Cu1	0.5000	0.0000	0.5000	0.02860 (13)	
O1	0.47417 (14)	0.2249 (2)	0.58090 (13)	0.0345 (4)	
O2	0.53762 (17)	0.4957 (2)	0.67150 (14)	0.0417 (4)	
O3	0.66880 (13)	0.1742 (2)	0.52517 (14)	0.0407 (4)	
O1W	0.42562 (15)	0.1187 (2)	0.33393 (14)	0.0374 (4)	
C1	0.5503 (2)	0.3554 (3)	0.61482 (17)	0.0325 (5)	
C2	0.6644 (2)	0.3444 (3)	0.5852 (2)	0.0376 (5)	
C3	0.7506 (2)	0.1723 (4)	0.4603 (2)	0.0442 (6)	
C4	0.8798 (2)	0.1745 (3)	0.54208 (19)	0.0341 (5)	
C5	0.9166 (2)	0.0908 (4)	0.6556 (2)	0.0427 (6)	
C6	1.0355 (3)	0.0908 (4)	0.7283 (2)	0.0510 (7)	
C7	1.1196 (2)	0.1751 (4)	0.6891 (2)	0.0513 (7)	
C8	1.0844 (2)	0.2568 (4)	0.5758 (3)	0.0507 (7)	
C9	0.9650 (2)	0.2583 (4)	0.5025 (2)	0.0430 (6)	
H1W1	0.425 (2)	0.2360 (13)	0.335 (2)	0.056*	
H1W2	0.457 (2)	0.075 (3)	0.286 (2)	0.056*	
H2A	0.6675	0.4486	0.5331	0.045*	
H2B	0.7333	0.3530	0.6601	0.045*	
H3A	0.7352	0.2803	0.4067	0.053*	
H3B	0.7361	0.0612	0.4094	0.053*	
H5A	0.8604	0.0339	0.6833	0.051*	
H6A	1.0593	0.0333	0.8045	0.061*	
H7A	1.1998	0.1768	0.7392	0.062*	
H8A	1.1414	0.3115	0.5482	0.061*	
H9A	0.9416	0.3157	0.4262	0.052*	

### Atomic displacement parameters (Å^2^)

**Table:**

	*U*^11^	*U*^22^	*U*^33^	*U*^12^	*U*^13^	*U*^23^
Cu1	0.0348 (2)	0.0229 (2)	0.03073 (19)	−0.00185 (16)	0.01464 (16)	−0.00319 (15)
O1	0.0435 (9)	0.0261 (8)	0.0393 (8)	−0.0028 (7)	0.0212 (7)	−0.0052 (7)
O2	0.0643 (11)	0.0267 (9)	0.0400 (8)	−0.0002 (8)	0.0254 (8)	−0.0066 (7)
O1W	0.0465 (10)	0.0316 (9)	0.0357 (8)	0.0020 (8)	0.0160 (7)	0.0004 (7)
C1	0.0444 (13)	0.0287 (12)	0.0245 (9)	0.0028 (10)	0.0122 (10)	0.0021 (9)
C2	0.0400 (13)	0.0300 (13)	0.0423 (12)	−0.0057 (10)	0.0136 (11)	−0.0086 (10)
O3	0.0369 (9)	0.0375 (9)	0.0537 (9)	−0.0061 (7)	0.0234 (8)	−0.0147 (8)
C3	0.0414 (14)	0.0559 (17)	0.0389 (12)	−0.0013 (12)	0.0188 (11)	−0.0086 (11)
C4	0.0382 (12)	0.0335 (12)	0.0347 (11)	0.0017 (10)	0.0178 (10)	−0.0028 (9)
C5	0.0493 (16)	0.0419 (14)	0.0427 (12)	0.0061 (12)	0.0233 (12)	0.0054 (11)
C6	0.0590 (18)	0.0537 (17)	0.0393 (13)	0.0205 (14)	0.0158 (13)	0.0053 (12)
C7	0.0400 (14)	0.0482 (17)	0.0584 (15)	0.0070 (13)	0.0077 (13)	−0.0118 (13)
C8	0.0413 (15)	0.0456 (16)	0.0726 (18)	−0.0036 (12)	0.0289 (14)	−0.0005 (14)
C9	0.0453 (14)	0.0432 (15)	0.0459 (13)	0.0038 (11)	0.0225 (12)	0.0055 (11)

### Geometric parameters (Å, °)

**Table:**

Cu1—O1	1.9420 (14)	C3—C4	1.500 (3)
Cu1—O3	2.2922 (14)	C3—H3A	0.9700
Cu1—O1W	2.0157 (15)	C3—H3B	0.9700
O1—C1	1.264 (3)	C4—C5	1.379 (3)
O2—C1	1.239 (2)	C4—C9	1.386 (3)
Cu1—O1^i^	1.9420 (14)	C5—C6	1.372 (4)
Cu1—O3^i^	2.2922 (14)	C5—H5A	0.9300
Cu1—O1W^i^	2.0157 (15)	C6—C7	1.374 (4)
O3—C3	1.424 (2)	C6—H6A	0.9300
O1W—H1W1	0.839 (9)	C7—C8	1.370 (4)
O1W—H1W2	0.83 (2)	C7—H7A	0.9300
C1—C2	1.512 (3)	C8—C9	1.380 (4)
C2—O3	1.414 (2)	C8—H8A	0.9300
C2—H2A	0.9700	C9—H9A	0.9300
C2—H2B	0.9700		
			
O1^i^—Cu1—O1	180.00 (7)	C1—C2—H2B	109.6
O1^i^—Cu1—O3	103.51 (6)	C2—O3—C3	114.88 (17)
O1—Cu1—O3	76.49 (6)	C2—O3—Cu1	110.48 (12)
O1^i^—Cu1—O1W	88.45 (6)	C3—O3—Cu1	130.94 (13)
O1—Cu1—O1W	91.55 (6)	C4—C3—H3A	108.9
O3—Cu1—O3^i^	180.0	C4—C3—H3B	108.9
O1W—Cu1—O3	88.11 (6)	C4—C5—H5A	119.7
O1W^i^—Cu1—O3	91.89 (6)	C4—C9—H9A	119.9
O1W—Cu1—O1W^i^	180.0	C5—C4—C9	118.8 (2)
Cu1—O1W—H1W1	114.0 (18)	C5—C4—C3	121.3 (2)
Cu1—O1W—H1W2	109.5 (18)	C5—C6—C7	120.4 (2)
O1^i^—Cu1—O3^i^	76.49 (6)	C5—C6—H6A	119.8
O1—Cu1—O3^i^	103.51 (6)	C6—C5—C4	120.6 (2)
O1^i^—Cu1—O1W^i^	91.55 (6)	C6—C5—H5A	119.7
O1—Cu1—O1W^i^	88.45 (6)	C6—C7—H7A	120.2
O1—C1—C2	119.44 (18)	C7—C6—H6A	119.8
O2—C1—O1	123.9 (2)	C7—C8—C9	120.3 (2)
O2—C1—C2	116.6 (2)	C7—C8—H8A	119.9
O3—C2—C1	110.35 (18)	C8—C7—C6	119.6 (2)
O3—C2—H2A	109.6	C8—C7—H7A	120.2
O3—C2—H2B	109.6	C8—C9—C4	120.3 (2)
O3—C3—C4	113.51 (18)	C8—C9—H9A	119.9
O3—C3—H3A	108.9	C9—C8—H8A	119.9
O3—C3—H3B	108.9	C9—C4—C3	119.9 (2)
O1W—Cu1—O3^i^	91.89 (6)	H1W1—O1W—H1W2	113.6 (15)
O1W^i^—Cu1—O3^i^	88.11 (6)	H2A—C2—H2B	108.1
C1—O1—Cu1	123.14 (13)	H3A—C3—H3B	107.7
C1—C2—H2A	109.6		
			
Cu1—O1—C1—O2	−176.53 (15)	O1W—Cu1—O3—C2	92.76 (14)
Cu1—O1—C1—C2	3.9 (3)	O1W^i^—Cu1—O3—C2	−87.24 (14)
Cu1—O3—C3—C4	−134.22 (17)	O1W—Cu1—O3—C3	−63.95 (19)
O1^i^—Cu1—O3—C2	−179.29 (13)	O1W^i^—Cu1—O3—C3	116.05 (19)
O1—Cu1—O3—C2	0.71 (13)	C1—C2—O3—C3	161.48 (18)
O1^i^—Cu1—O3—C3	24.0 (2)	C1—C2—O3—Cu1	0.7 (2)
O1—Cu1—O3—C3	−156.0 (2)	C2—O3—C3—C4	69.9 (3)
O1—C1—C2—O3	−2.8 (3)	C3—C4—C5—C6	178.6 (2)
O2—C1—C2—O3	177.60 (17)	C3—C4—C9—C8	−178.3 (2)
O3—Cu1—O1—C1	−2.51 (15)	C4—C5—C6—C7	0.3 (4)
O3^i^—Cu1—O1—C1	177.49 (15)	C5—C4—C9—C8	0.2 (3)
O3—C3—C4—C5	31.9 (3)	C5—C6—C7—C8	−1.2 (4)
O3—C3—C4—C9	−149.7 (2)	C6—C7—C8—C9	1.5 (4)
O1W—Cu1—O1—C1	−90.19 (16)	C7—C8—C9—C4	−1.0 (4)
O1W^i^—Cu1—O1—C1	89.81 (16)	C9—C4—C5—C6	0.1 (3)

Symmetry codes: (i) −*x*+1, −*y*, −*z*+1.

### Hydrogen-bond geometry (Å, °)

**Table:**

*D*—H···*A*	*D*—H	H···*A*	*D*···*A*	*D*—H···*A*
O1W—H1W1···O2^ii^	0.839 (9)	1.978 (12)	2.796 (2)	165 (2)
O1W—H1W2···O2^iii^	0.83 (2)	1.964 (10)	2.788 (2)	173 (2)

Symmetry codes: (ii) −*x*+1, −*y*+1, −*z*+1; (iii) *x*, −*y*+1/2, *z*−1/2.

## References

[bb1] Bruker (2004). *APEX2* and *SAINT* Bruker AXS Inc., Madison, Wisconsin, USA.

[bb2] Eddaoudi, M., Chen, B., O’Keeffe, M. & Yaghi, O. M. (2005). *J. Am. Chem. Soc.* **127**, 1504–1510. 10.1021/ja045123o 15686384

[bb3] Sheldrick, G. M. (1996). *SADABS* University of Göttingen, Germany.

[bb4] Sheldrick, G. M. (2008). *Acta Cryst.* A**64**, 112–122. 10.1107/S0108767307043930 18156677

